# Metabolic modeling predicts specific gut bacteria as key determinants for *Candida albicans* colonization levels

**DOI:** 10.1038/s41396-020-00848-z

**Published:** 2020-12-15

**Authors:** Mohammad H. Mirhakkak, Sascha Schäuble, Tilman E. Klassert, Sascha Brunke, Philipp Brandt, Daniel Loos, Ruben V. Uribe, Felipe Senne de Oliveira Lino, Yueqiong Ni, Slavena Vylkova, Hortense Slevogt, Bernhard Hube, Glen J. Weiss, Morten O. A. Sommer, Gianni Panagiotou

**Affiliations:** 1 Systems Biology & Bioinformatics Unit, Leibniz Institute for Natural Product Research and Infection Biology - Hans Knöll Institute, 07745 Jena, Germany; 2 ZIK Septomics, Jena University Hospital, 07745 Jena, Germany; 3 Department of Microbial Pathogenicity Mechanisms, Leibniz Institute for Natural Product Research and Infection Biology - Hans Knöll Institute, 07745 Jena, Germany; 4 Septomics Research Center, Friedrich Schiller University, Leibniz Institute for Natural Product Research and Infection Biology - Hans Knöll Institute, 07745 Jena, Germany; 5 Novo Nordisk Foundation Center for Biosustainability, Technical University of Denmark, 2800 Kgs. Lyngby, Denmark; 6 Institute for Microbiology, Friedrich Schiller University, 07743 Jena, Germany; 7 MiRanostics Consulting, 85755 Oro Valley, AZ USA

**Keywords:** Fungi, Infectious diseases, Metagenomics, Gastrointestinal diseases, Microbiome

## Abstract

*Candida albicans* is a leading cause of life-threatening hospital-acquired infections and can lead to Candidemia with sepsis-like symptoms and high mortality rates. We reconstructed a genome-scale *C. albicans* metabolic model to investigate bacterial-fungal metabolic interactions in the gut as determinants of fungal abundance. We optimized the predictive capacity of our model using wild type and mutant *C. albicans* growth data and used it for in silico metabolic interaction predictions. Our analysis of more than 900 paired fungal–bacterial metabolic models predicted key gut bacterial species modulating *C. albicans* colonization levels. Among the studied microbes, *Alistipes putredinis* was predicted to negatively affect *C. albicans* levels. We confirmed these findings by metagenomic sequencing of stool samples from 24 human subjects and by fungal growth experiments in bacterial spent media. Furthermore, our pairwise simulations guided us to specific metabolites with promoting or inhibitory effect to the fungus when exposed in defined media under carbon and nitrogen limitation. Our study demonstrates that in silico metabolic prediction can lead to the identification of gut microbiome features that can significantly affect potentially harmful levels of *C. albicans*.

## Highlights

- Genome-scale model reconstruction of *C. albicans* with 89% growth accuracy.
- Mutualism and parasitism are the most common predicted *C. albicans*-gut bacteria interactions.
- Metagenomic sequencing and in vitro assays reveal modulators of fungal growth.
- *Alistipes putredinis* potentially prevents elevated *C. albicans* levels.

## Introduction

The fungus *Candida albicans* is found on the mucosal surfaces of at least 50–70% of healthy adults [1] and is a classic opportunistic pathogen. It resides as a harmless commensal but can become pathogenic in immunocompromised patients or under microbial dysbiosis [2, 3]. *C. albicans* causes up to 300,000 deaths per annum worldwide with an increasing number of individuals at risk [4]. Therefore, efforts to understand drivers of commensal or pathogenic outcomes of this fungus have intensified.

Recent studies found links between alterations in the composition and functionality of the gut microbiota and development of local or systemic *C. albicans* infections [5, 6]. A microbial tryptophan metabolic pathway appears to preserve immune physiology at mucosal surfaces by promoting indole-3-aldehyde production that contributes to IL-22 transcription [7]. Other gut microbial products such as bacteriocin are directly active against *C. albicans* [8]. A study of rectal samples from a cohort of 150 children linked gut microbiota to *Candida* prevalence, with a relative reduction in *Candida* species in children who received probiotics along with broad spectrum antibiotics [9]. Neither the dynamics of *Candida* species in the human gut nor the specific microbial contributors to the observed reduction have been studied, however. Based on these diverse findings, the commensal status of *C. albicans* appears to be related to the global taxonomy and functionality of the host microbiome.

A promising approach to analyzing interactions between *C. albicans* and gut microbial species uses genome-scale metabolic models (GSMMs) GSMMs have improved the biotechnological productivity of bacteria [10–12], revealed plant metabolic processes [13], and elucidated the Crabtree effect in yeast [14–16] and the Warburg effect in cancer cells [17]. Recent pioneering studies have developed high-quality GSMMs for gut bacteria that enable in silico analysis of gut metabolic functions and interactions [18, 19]. These resources have advanced the study of gut microbes and their respective pairwise interactions but have not yet been used to study interactions with opportunistic fungal pathogens such as *C. albicans*. The potential of gut microbes to influence the overall fitness of the fungus must be elucidated to support development of prophylactic or therapeutic strategies to control *C. albicans*.

We constructed a GSMM of *C. albicans*, starting with an automatically generated template model [20, 21]. We substantially improved its performance with manual curation and adaptation to phenotype microarray experiments. We used both, publicly available data [22] and new phenotypic microarray data for both wild type and mutant *C. albicans* strains. Our model predictions surpassed those of other GSMMs for species closely related to *C. albicans* that could serve as proxies for this fungus. We used the GSSM to simulate in silico pairwise metabolic interactions between *C. albicans* and each of 910 gut bacteria models. We challenged our predictions in vitro by growing *C. albicans* in carbon or nitrogen limited defined media in the presence of predicted fungal growth affecting metabolites. We further validated our results with stool samples from 24 human subjects, using metagenomics and internal transcribed spacer (ITS) sequencing to identify bacterial species associated with significant effects on *C. albicans* metabolism and growth. Finally, we assessed fungal growth in bacterial spent media experiments.

## Materials and methods

### Model reconstruction

**Fig. 1:**
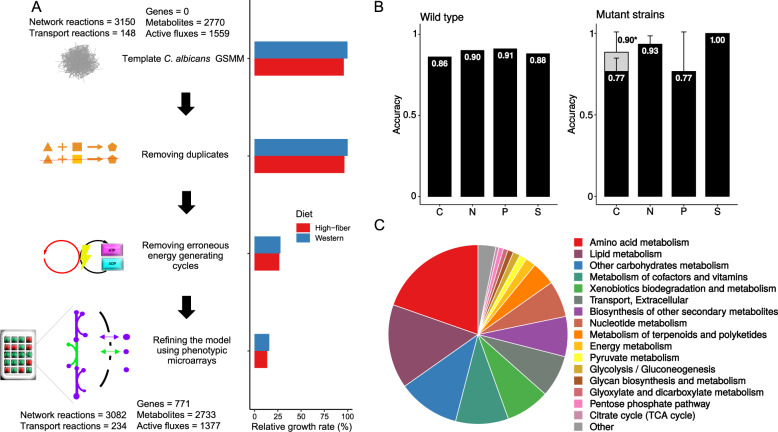
*Candida albicans* GSMM reconstruction. **A** Based on a template [20] a manually curated metabolic model was achieved in several steps. Adjustment of model features such as modifying metabolic reactions or resolving energy cycles and model impact are indicated. Relative growth rates show relative differences to the template GSMM growth rate. **B** Benchmark results for model optimization using phenotypic microarray data for *C. albicans* growth. Light gray bar indicates accuracy on carbon media without arginine mutants that show growth on phenotypic microarray data without additional arginine (see main text for details). Accuracy was calculated as number of growth experiments that agree with model predictions across all growth experiments that were simulated. C carbon, N nitrogen, P phosphor, S sulfur. **C** Assigned pathway distribution of the final model.

To generate the *C. albicans* GSMM, we used the *C. albicans* metabolic model automatically reconstructed by the CoReCo pipeline as a template. In brief, CoReCo combines information from multiple data sources into a unified database and evaluates the probability of any reaction occurring in the target organism by computing a score for each enzyme based on sequence homology [20]. We refined the model in four consecutive steps (Fig. 1A) that included the identification and removal of duplicate metabolites, determination and resolve of erroneous energy-generating cycles [23], adaptation to phenotypic microarray data and exchange reaction modification based on flux variability analysis (FVA, Supplementary Material, Supplementary Data S1) [24].

### Pairwise simulations

Pairwise simulations adapted from Heinken and Thiele [25] were performed using 818 AGORA 1.03 GSMMs [18] downloaded from (https://www.vmh.life) and 92 CarveMe gut bacterial GSMMs [19]. In brief, the *C. albicans* GSMM was paired to individual bacterial GSMMs and subsequently optimized by simultaneously maximizing *C. albicans* and bacteria biomass reactions. Interaction type was determined by taking the optimized growth rate in the pair compared to the growth rate of the individual GSMM into account (Supplementary Material).

To evaluate dissimilarities between promoters and inhibitors of *C. albicans* from different phyla, Bray-Curtis distances were calculated based on flux distributions of individual bacteria from pFBA simulations on different media [18] (https://www.vmh.life, Supplementary Table S1). Fisher’s exact tests were calculated to determine if a specific phylum was enriched with species that either inhibited or promoted *C. albicans* growth. The python code and metabolic models for simulating pairwise GSMMs are available at https://github.com/mohammadmirhakkak/Candida-albicans-microbiome-interaction.

### Phenotypic microarray experiments

*C. albicans* wild type and multiple mutant strains (Table 1) were pre-grown on YPD (1% yeast extract, 2% peptone, 2% glucose, 2% agar) plates. Phenotype microarrays were performed by using microarray plates, reagents and devices according to the manufacturer Biolog, Inc. (Hayward, CA, USA) instructions. Briefly, *C. albicans* cells were taken directly from YPD agar plates and diluted in sterile dH2O to 62% transmittance as measured by a turbidimeter (Biolog, Inc.). Next, cells were combined with inoculating fluid IFY-0 base (1.2x), redox dye mix D (75x) (Biolog Inc.), and further supplemented with either Glucose, L-glutamic acid, potassium phosphate or sodium sulfate (Sigma-Aldrich) were required. 100 µl of the respective mixture (83.33% IFY-0 base, 1.33% redox dye D, 8.33% supplements, 2.08% cells, and if required 3.12% Glucose and dH2O) was added to each well of a Biolog Phenotype Microarray 96-well plate for fungi to test for metabolic activity in the presence of carbon sources (PM1 and PM2A), nitrogen sources (PM3B), and phosphorus and sulfur sources (PM4A). The phenotype microarrays were incubated at 30 °C in an OmniLog multiple plate reader in order to prevent hyphae formation that otherwise perturb growth measurements. Reduction of the redox dye, an indicator for metabolic activity, was measured kinetically every 15 min at an OD of 750 nm for 24–48 h. Each experiment was performed twice. Data analysis was done using the R opm package for OmniLog phenotype microarray datasets [26].

**Table 1:** Selected *Candida albicans* strains for phenotypic microarrays experiments.

Strain	Genotype	Reference
SC5314	Prototroph	[65]
CEC2908	*ura3∆::λimm434/ura3∆::λimm434 his1∆::hisG/HIS1 arg4∆::hisG/ARG4 ADH1/adh1::pTDH3-carTA::SAT1*	[66]
SN87	*leu2Δ/leu2Δ his1Δ/his1Δ URA3/ura3Δ::imm434 IRO1/iro1Δ::imm434*	[67]
SN152	*leu2Δ/leu2Δ +LEU2, his1Δ/his1Δ +HIS1, arg4Δ/arg4Δ, URA3/ura3Δ::imm434, IRO1/iro1Δ::imm434*	[68]
JRC12	*arg1Δ::FRT/arg1Δ::FRT*	[69]
JRC38	*arg3Δ::FRT/arg3Δ::FRT*	[69]
CFG318	*NEUT5L/neut5l::FRT*, *put2Δ/put2Δ*	[47]

### In vitro experiments

#### Growth of C. albicans in presence of metabolites

To determine the effect of the metabolites on *C. albicans* growth, the clinical isolate SC5314 was grown overnight at 30 °C in YPD complex medium (1% yeast extract, 2% peptone, 2% glucose). 30 °C were chosen to prevent hyphae formation provoked at higher temperatures, which otherwise perturb growth curve measurements. Yeast cells were washed three times with sterile H_2_O by centrifugation for 5 min at 4,200 × *g*. Test medium was composed of 1× yeast nitrogen base (YNB, Formedium) with either (standard) 0.25% NH_4_SO_4_/2% glucose, (C limited) 0.25% NH_4_SO_4_/0.25% glucose, (N limited) 0.008% NH_4_SO_4_/2% glucose, or (C/N low) 0.016% NH_4_SO_4_/0.5% glucose. Test substances were obtained from Sigma-Aldrich and were dissolved in H_2_O at (nitrite) 156 mM, (desoxy-adenosine) 100 mM, (sodium formate) 1 M, (putrescine) 500 mM, (L-asparagine) 100 mM, or (L-proline) 15.6 mM. Assays were performed as 1:2 dilution series in 96 well plates (TPP, flat bottom) and were composed of 180 µl test medium, 10 µl test substance, 10 µl yeast solution (1:10 dilution in H_2_O, final OD_600_ of 0.1). Initial pH was verified to be at the expected ≈5.8. Growth was followed over at least 24 h using a Tecan infinite 200 multiwell plate reader set to 30 °C, with measurements at 600 nm every 15 min following 10 s of orbital shaking. All measurements were performed in triplicate from independent overnight cultures at different days. Growth was evaluated as area under the curve (AUC, trapezoidal method using GraphPad prism 8.1.2; baseline at mean of first three measurement) over 24 h and expressed as percent change compared to control setups (H_2_O instead of test substance) in the same medium. AUCs were determined for three replicates, and mean change compared to controls is shown with standard deviations (SD) as error bars.

#### Strains and culture conditions

*A. putredinis* (DSM17216), *B. ovatus* (ATCC 8483), *B. vulgatus* (ATCC8482), *E. lenta* (DSM2243), *P. copri* (DSM18205), *R. torques* (ATCC27756), *E. coli* (MG1655 and BAA-1161), *P. corporis* (DSM18810), *C. albicans* (SC5314/ATCC MYA-2876), *C. albicans* (ATCC 10231) and *C. albicans* (ATCC 18804) were grown at 37 ˚C under anaerobic conditions (gas mixture, 95% N_2_ and 5% H_2_) in prereduced modified GAM (mGAM, Nissui Pharmaceutical Co. Ltd.) broth for liquid cultures or broth supplemented with agar for growth on plates. 37 ˚C and anaerobic conditions were chosen to resemble best the natural environment of these gut bacteria.

#### Sterile bacterial spent media

Bacterial strains were grown for 48 h in GAM broth, then subcultured 1:50 in fresh GAM broth and grown for 48 h in anaerobic conditions at 37 °C to resemble the gut environment. Cultures were centrifuged at 11,000 × *g* for 5 min and spent media removed without disturbing the pellet. Spent media were passed through a 0.2 µm syringe filter to remove remaining bacteria. After filtration, the pH of the spent media was analyzed using an electronic pH-meter (Supplementary Table S2). 1% (v/v) of Phosphate-buffered saline (PBS) was added in each experiment to maintain a constant pH.

#### Growth assays

An overnight culture of *C. albicans* was grown aerobically in mGAM media at 37 °C. Aerobic conditions were chosen to enable sufficient growth of *C. albicans*. Cells were then subcultured at a 1:1000 dilution into 150 μl of sterile spent bacterial media in different proportions: 75 and 100%. The spent media were diluted in fresh 25% mGAM broth for 75% spent media proportion and PBS (1% v/v), accordingly. The fermentation was performed in flat-bottom, 96-well plates. The plates were incubated for 24 h at 37 °C, with continuous orbital shaking at 900 rpm. 37 °C did not induce notable hyphae formation in growth assays and therefore did not perturb growth measurements on spent media. Cell densities were measured every 10 min at optical density 600 nm (OD600) using a microtiter reader (BioTek ELx800). Growth rates were calculated by plotting log of OD measurements in log phase and calculating the slope of the time points in log phase where *r*^2^ was closest to 1, using at least 12 time points over 2 h. Growth inhibition was determined as the growth rate of *C. albicans* in spent bacterial media normalized to the growth rate in the corresponding mGAM fresh media dilution.

### Microbiome profiling

Bacterial and fungal species profiles were generated for stool samples from a human cohort of 24 individuals at Western Regional Medical Center, Goodyear, Arizona, USA. Samples were collected after signed informed consent under a protocol approved by the Western Institutional Review Board (WIRB protocol number 20140271, Pallyup, Washington, USA). All subjects had been diagnosed with different types of cancer, with heterogeneous stage, treatment, and histological findings. Metagenomic sequencing was performed at BGI, Hong Kong S.A.R., China, as described in Qin et al. [27], and ITS amplicon sequencing of the mycobiome was performed at ZIK Septomics, University Hospital Jena, Thuringia, Germany (extended details in Supplementary Material). Bacterial reads were obtained using 150-bp Illumina PE whole metagenome sequencing. Species profiling was by MetaPhlAn 2.7.6 [28]. after applying an in-house pipeline for quality control [29] and removal of human reads. Fungal reads were obtained using 250-bp Illumina PE ITS1 amplicon sequencing (extended details in Supplementary Material). The DADA2 ITS Pipeline Workflow 1.8 was followed for amplicon sequence variants [30]. Mothur classifier [31] called from QIIME 1.9.1 [32] and the UNITE database 7.2 [33] were used for fungal taxonomic assignments. Bacterial species were considered for further analysis, if a GSMM model was available, the species was part of the Open Tree of Life 10.4 [34] and it was prevalent in at least 4 of 26 samples. Correlations of bacterial abundance and growth rates to *C. albicans* abundance were tested using two-sided Spearman correlation (*p* < 0.05). The bacterial and fungal profiles are available under the ENA Study Nr. PRJEB33756.

Partial spearman correlation was computed within R using the package PResiduals (v0.2–6).

### Ordinal regression model

Relative abundance values for *C. albicans* from human samples were grouped into two sets determined by a 20% relative abundance threshold. To predict *C. albicans* abundance levels, three binomial ordinal regression models were generated using as independent variables the bacterial abundance values, predicted interaction coefficients, and products of multiplying abundance values by predicted interaction coefficients. For model building, we either selected five bacterial species that significantly correlated with *C. albicans* by relative abundance and two that significantly correlated by GRiD value, or selected bacteria based on whether they increase performance as described in the main text. The predictive power of each model was assessed by determining the true/false positive/negative and accuracy values and by analyzing receiving operating characteristic curves using the R package ROCR (ver. 1.0–7).

## Results and discussion

### Reconstruction of a *Candida albicans* genome-scale metabolic model

To develop a *C. albicans* GSMM, we started with a model automatically generated by the Comparative ReConstruction (CoReCo) pipeline [20]. The initial *C. albicans* CoReCo model (BioModels ID 1604280052) comprised 2770 metabolites and 3298 reactions, of which 3150 were network and 148 transport reactions (Fig. 1A). In addition to a unique reaction set, the initial CoReCo GSMM for *C. albicans* contained multiple nontrivial duplicate reactions and metabolites. Typically, these involved marginally differing metabolite names such as *L-Glutamate* and *Glutamate* that had not been automatically detected by the CoReCo platform (Fig. 1A, Supplementary Data S1, Tables S1, S3, and S4).

We also curated energy-generating cycles (EGCs) that created energy compounds such as ATP without requiring nutrient uptake [23]. We resolved these infeasible EGCs by identifying and correcting implausible reaction directionalities using metabolic pathway databases such as BioCyc (Supplementary Table S4). For example, in our initial *C. albicans* model, we found an ATP-producing EGC that involved phosphate rather than pyrophosphate as indicated by BRENDA, Biocyc and KEGG databases. Correcting the involved reaction acetoacetate:CoA ligase resolved this particular EGC and additional EGCs while maintaining a viable biomass flux. We corrected six reactions by either changing metabolite usage or reaction directionality based on KEGG or BioCyc information (Supplementary Table S4). Resolving EGCs also reduced the flux through the biomass reaction towards 1.4, which is closer to the biomass reaction flux for other fungal models such as the yeast consensus model [35, 36] (Fig. 1A).

Next, we adapted our model to multiple phenotypic microarray growth experiments of *C. albicans*. These comprise up to 1440 different defined media experiments with diverse carbon, nitrogen, phosphorus or sulfate sources. We used a publicly available dataset with different *C. albicans* phenotypes [22] and created additional phenotypic microarray growth experiments including several mutant strains for e.g., different arginine biosynthesis steps (see Methods for details). Of the metabolites in the dataset, 455 mapped to metabolites in our *C. albicans* GSMM. First, we adapted our model to growth experiments that were in agreement between data from Ene et al. [22] and our own prototrophic *C. albicans* wild type strain SC5314 dataset for *C. albicans*. Of note, using only compatible growth measurements ensured robust growth information across different temperatures applied in our and in the published data by Ene et al. [22] (cf. Materials and Methods). Second, we adapted our model to all phenotypic growth experimental data of *C. albicans* mutant strains for those growth conditions that yielded the same results for both our wild type and the Ene et al. dataset in the prior step (Supplementary Table S4). These refinement steps included, for example, enabling ammonia production from urea or via lysine degradation, which was not initially present in our model (Supplementary Tables S4 and S5). Based on the growth data, we added 107 potentially feasible exchange reactions involving metabolites associated with *C. albicans* growth. By applying flux variability analysis [24] we also identified five exchange reactions that shuttle 4-aminobenzoate, folinic acid, 7,8-diaminononanoate, hexadecanal, and hydrogensulfite in and out of the network, but do not support growth and removed these from the GSMM accordingly. Overall, we achieved a high compatibility between our model predictions and the phenotypic microarray growth experiments with nitrogen and sulfur predictions reaching above 90% accuracy over all mutant experiments (Fig. 1B, Supplementary Table S4). Of note, model predictions for carbon source experiments of three different *C. albicans* mutants for arginine biosynthesis (*arg1*Δ, *arg3*Δ and *arg4*Δ) predicted no growth rate for all carbon sources, since arginine is essential for the biomass objective function. Surprisingly, up to 33% of the associated phenotypic microarray data showed fungal growth, despite the inability to synthesize arginine due to the knock out. This might be due to recycling of available proteins in e.g., fungal vacuoles, as the defined growth media itself does not contain arginine, unless specifically tested. Considering only growth experiments that show no growth for arginine biosynthesis mutant strains our model accuracy for carbon sources reaches 90% across all tested *C. albicans* mutant (Fig. 1B).

Finally, we added gene annotation for ~1500 reactions and associated individual gene to reaction rules using the KEGG database. We also added pathway association for all reactions if available and unified pathway associations across resources. This step resulted in 83 pathway definitions, including all essential pathways such as in central carbon, amino acid, and lipid metabolism (Fig. 1C). Overall, our model refinements resulted in an addition of 771 genes, led to a final model comprising 3082 metabolic reactions (−68 compared to draft model) and 2733 metabolites (−37 metabolites compared to draft model) and a reduced active flux flexibility towards biomass by 11.7% (Fig. 1A, Supplementary Data S1, Tables S1, S3, and S4).

### Pairwise growth simulations predict gut bacteria modulating essential *C. albicans* metabolic activity

Next, we generated in silico metabolic interaction predictions about *C. albicans* coupled to gut bacteria GSMM models. Using 910 publicly available GSMMs for gut microbial species [18, 19], we performed pairwise metabolic analysis by linking our *C. albicans* model with each gut microbe GSMM [25] (Supplementary Table S1). The majority of GSMMs from different sources [18, 19] gave compatible growth rate predictions. To streamline further analysis, we continued with the assembly of gut organisms through reconstruction and analysis (AGORA) GSMMs (https://www.vmh.life) [18] unless models were available from both sources. In the latter case, we continued with the CarveMe model versions, since these are refined to bacterial growth data across 19 different media including extended pathway gap correction [37] and are based on a manually curated template model [19].

**Fig. 2:**
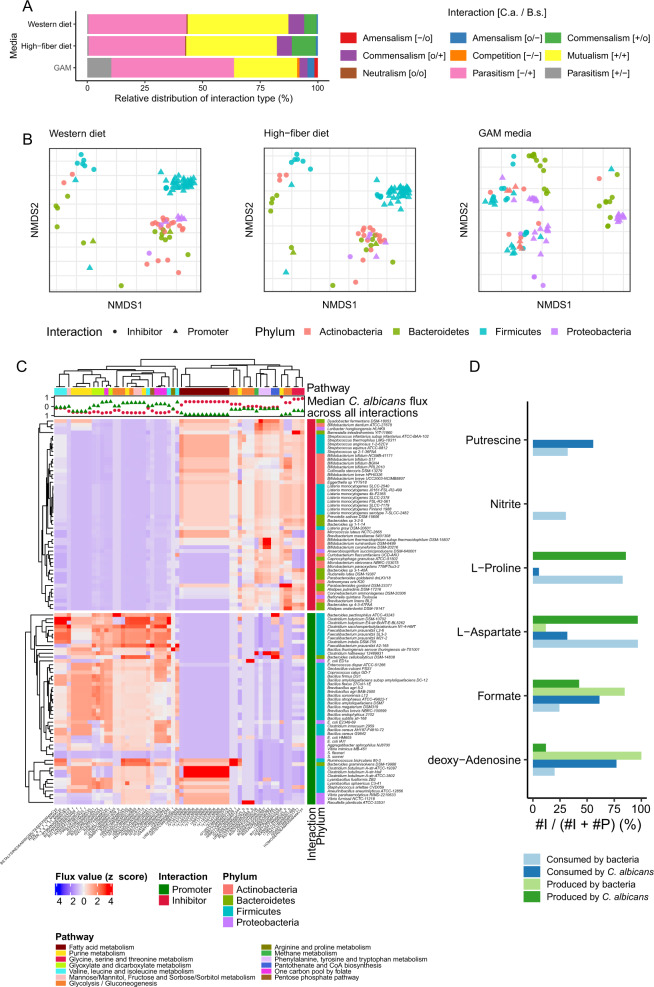
Pairwise in silico interaction experiments. **A** Distribution of interaction type for *C. albicans* (C.a.) and bacterial species (B.s.). Interactions have positive (+), negative (−) or no (o) effect on growth rates of fungus or bacteria as indicated for interaction types. **B** Non-metric multidimensional scaling (NMDS) plots of bacterial reaction flux rates for top 50 *C. albicans-*inhibiting and -promoting bacteria simulated for three different media (Western and high-fiber diet, Gifu anaerobic media (GAM)). **C** Metabolic reactions of *C. albicans* with the most substantially differing flux rates of *C. albicans* when paired with top 50 inhibiting or promoting bacteria. Top: median *C. albicans* flux rate differences across all bacterial species paired with *C. albicans*. **D** Analysis for selected metabolites based on exchange reaction fluxes of simulated fungal–bacterial pairs for top 50 promoting or inhibiting bacterial species (cf. Supplementary Table S5). *x*-axis indicates the percentage of exchange reaction fluxes with bacteria that inhibit *C. albicans* growth.

We simulated growth on two different media compositions that resemble typical Western and high-fiber diets (https://www.vmh.life) [18]. We identified the interaction type of each *C. albicans*-gut microbe pair by analyzing differences in predicted growth rates compared to growth rates derived from individual simulations using a flux balance analysis approach [38] (Methods, Pairwise simulations; Supplementary Material, Table S1). Predictions based on Western and high-fiber diets gave similar interaction-type distributions: mutualism (positive growth effect for both, *C. albicans* and paired bacteria) and parasitism (here, negative effect on *C. albicans* growth, positive growth effect on bacteria) were the most abundant (>81% of observed interaction types, Fig. 2A). Other interaction predictions included commensalism, in which both *C. albicans* and bacterial growth was promoted without negative effects on the respective paired microorganism (12% and 17% for Western and high-fiber diets, respectively). Only a few examples of parasitism in which *C. albicans* exerted negative effects on gut bacteria or amensalism (no growth effect on *C. albicans*, negative effect on bacteria) or neutralism (no growth effect on both, *C. albicans* and gut bacteria) were observed. We further examined prediction accuracy with additional simulations on standard Gifu anaerobic medium (GAM), which was used for in vitro validation experiments (Supplementary Table S6). Growth simulations on GAM were feasible for only 200 GSMM models, as all other models yielded no biomass formation. GAM simulations identified parasitism with negative effects on bacteria for up to 16% of viable bacteria models, while parasitism with negative effects on *C. albicans* (53%) and mutualism (28%) were again the most commonly occurring interaction types (Fig. 2A). Our interaction-type analysis based on predicting growth rate differences for microorganisms in individual and paired model setups hints that the majority of gut bacteria have either a mutualistic or a parasitic relationship with *C. albicans* irrespective of medium.

To analyze if predicted interactions that promoted or inhibited *C. albicans* growth were phylum- or diet-specific effects, we performed non-metric multidimensional scaling analysis (NMDS) based on parsimonious flux balance analysis (pFBA)-derived values for the top 50 promoting and inhibiting bacterial species [39] (Supplementary Table S1). The predicted top 50 inhibiting bacterial species comprised mainly species from the genus Bifidobacterium and Listeria, but also included several bacteria from the Bacteriodetes phylum including e.g., *Alistipes putredinis*. In contrast, the predicted top 50 *C. albicans* promoting bacteria included many bacteria with the Bacillus, Bacteroides, Clostridium or Vibrio genus (Supplementary Table S1). After removal of a few outliers (up to seven GSMMs comprising several *Escherichia coli* strains, among others) we observed distinct grouping of the Firmicutes phylum on both Western and high-fiber diets in the NDMS plots, with a largely predicted positive effect on *C. albicans* growth (Fig. 2B). The majority of Proteobacteria (for 71% of the respective GSMMs on Western, and 73% on high-fiber diet) were predicted to show a positive effect on *C. albicans* growth as well and show similar flux distributions to some Bacteroidetes and most Actinobacteria. In contrast, the predicted impact of Bacteroidetes was primarily negative (for 87% GSSMs on Western, and 86% on high-fiber diet) on *C. albicans* growth with differing flux distributions for both, Western and high-fiber diet (Fig. 2B). All Actinobacteria were predicted to inhibit *C. albicans* growth as well and showed similar flux distributions to some Bacteroidetes and most Proteobacteria for Western and high-fiber diets. Of note, in silico simulations on GAM revealed notable differences of flux distributions compared to Western or high-fiber diet (e.g., 53% of the paired Actinobacteria showed promoting effects on *C. albicans*) showing that GAM based simulations differ to some degree from these diets (Fig. 2B). The predicted interaction type was significantly dependent on phyla (chi-squared test, *p* value = 2.5e−9 on Western diet) with Actinobacteria (false discovery rate-corrected Fisher’s exact test *p* value = 7.0e−7) and Firmicutes (false discovery rate-corrected Fisher’s exact test *p* value = 1.5e−5) primarily responsible for the interaction type. Altogether, our analyses suggested that under Western and high-fiber diets, species from specific phyla had positive (Firmicutes, Proteobacteria), or negative (Actinobacteria, Bacteriodetes) effects on *C. albicans* growth with distinct flux distributions particularly for predicted growth-promoting Firmicutes species.

Next, we investigated *C. albicans*-specific reaction fluxes derived from paired fungal–bacterial model simulations. Paired bacteria were again selected based on their predicted impact on *C. albicans* growth rates such that its difference between paired fungal–bacterial and individual model simulations is most pronounced and comprised bacteria from four different phyla (Fig. 2C, Supplementary Fig. S1). From the obtained flux distributions *C. albicans* reactions were selected based on the most pronounced flux differences between *C. albicans* paired with predicted growth inhibiting and growth-promoting bacterial GSMMs (Methods, Pairwise simulations). Of note, predicted median flux differed notably for many reactions comparing the top inhibiting and promoting bacteria to all paired gut microbes (Fig. 2C, upper panel). These changes were particularly present in sugar, fatty acid, folate, and small amino acid associated pathways, but also in glycolysis and hint towards a shifted flux in the selected bacteria that dominantly affect *C. albicans* growth. Altered reaction fluxes were identified across major pathways including carbon, amino acid, purine and fatty acid metabolism. Central metabolites such as alpha-ketoglutarate, pyruvate and glutamate were balanced towards net production or consumption, depending on the paired gut microbe in the in silico simulation. *C. albicans* growth is affected by modulating carbohydrate metabolism [40] or the availability of amino acids such as leucine or valine [41] and might be used by gut microbiota to prevent or promote *C. albicans* growth. Specifically, we predicted elevated reaction fluxes that consumed L-glutamate in *C. albicans* when paired with fungal growth-promoting bacteria via the aminotransferases Glycine:2-oxoglutarate aminotransferase and branched-chain amino acid aminotransferase. Aminotransferases were studied before in the context of Candida infection and were attributed to the nutritional versatility of Candida species [42, 43]. Moreover, glutathione synthesized from L-glutamate is important in counteracting oxidative stress [44]. Predicted reaction fluxes including amino acids were notably different among top promotors and inhibitors and may serve as potential targets for identifying antifungal agents [45]. These findings suggest that gut bacteria that potentially perturb *C. albicans* growth may cause metabolic shifts in *C. albicans* towards L-glutamate-promoting reactions, which may allow the fungus to evade harmful oxidative stress levels.

Next, we focused on investigating fluxes of metabolite exchange reactions. Exchange reactions allow to shuttle metabolites in and out of a joint compartment in our paired in silico models. These joint compartments serve as a connection between the respective bacterial and the fungus model and allow to predict potentially *C. albicans* growth rate influencing metabolites (Fig. 2D, Supplementary Table S7). To identify such metabolites we specifically filtered for exchange reaction fluxes of metabolites that are primarily present for either *C. albicans* growth inhibiting or promoting bacteria as derived from our paired fungal–bacterial model simulations. Again, many amino acids like L-proline, or L-aspartate, but also other factors such as nitrite or putrescine are predicted to be notably differentially consumed by fungus or bacteria.

### In vitro experiments and metagenomics analyses support metabolic dependencies of *C. albicans*

To test the quality of our in silico analysis, we investigated *C. albicans* growth in the presence of metabolites, performed metagenomic sequencing for 24 individuals and assessed *C. albicans* growth in bacterial spent media.

**Fig. 3:**
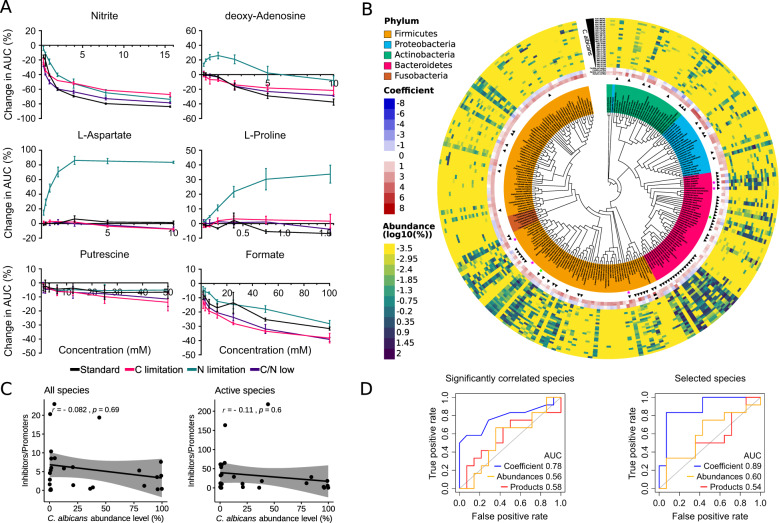
Experimental and clinical data supporting in silico predictions. **A** Area under the curve (AUC) measurements for fungal growth in presence of selected metabolites in a series of concentration dilution experiments. AUCs were determined for three replicates. Mean change compared to medium-only controls is shown with standard deviations (SD) as error bars. **B** Bacterial abundance and growth rates were obtained using MetaPhlAn2 and GRiD 1.2, respectively. Modeled species were arranged according to the Open Tree of Life 10.4 [34]. Annotation rings from inner to outer: Significant correlations between *C. albicans* abundance and bacterial abundance (magenta stars) or bacterial GRiD score (green stars, Spearman’s coefficient, *p* < 0.05); species with GRiD score greater than 1 in at least one sample (black triangles); in silico interaction coefficients from GSMM analysis (blue to red); sample bacterial abundance (*N* = 26, yellow to purple) sorted by *C. albicans* abundance (highest abundance is outermost ring). **C** Regression performance for all (left panel) or active (GRiD value > 1 in at least one patient, right panel) species. Each dot is a ratio of inhibitors to promoters for a patient. Values for inhibitors and promoters were calculated by summing products of bacterial abundance x in silico coefficient for *C. albicans*. **D** Area under the curve (AUC) of ordinal regression analysis for seven species with significant correlations with *C. albicans* abundance or GRiD values as shown in **B** (left panel). AUC of ordinal regression analysis for 29 selected species (see main text). The regression model performance was achieved by using GSMM based metabolic coefficients (Coefficients), bacterial relative abundance (Abundances) or the product of both (Products).

First, we grew *C. albicans* in the presence of metabolites and investigated the growth-promoting or -inhibiting effect of these metabolites under different carbon and/or nitrogen availabilities (Fig. 3A). We selected six metabolites that were either primarily consumed or produced by either fungus or bacteria in our paired metabolic in silico simulations (Fig. 2D, Supplementary Table S7). We hypothesized that metabolites that e.g., are predicted to be consumed by fungal growth inhibiting bacteria are either withheld from the fungus or cannot be metabolized by the fungus and are thus beneficial for the bacteria to outgrow *C. albicans*. Likewise, metabolites that are predicted to be produced by bacteria or consumed by the fungus when *C. albicans* is paired with growth inhibiting bacteria might provide clues of metabolites with a potential negative effect on *C. albicans* growth. Nitrite showed severe growth inhibiting effects on *C. albicans*, irrespective of available carbon and nitrogen source concentrations. The same inhibiting influence was observed to a lesser extent for putrescine and for the formic acid salt, sodium formate. Both putrescine and formic acid were also predicted to be primarily consumed by *C. albicans* when paired with fungal growth inhibiting bacteria. In contrast, L-Aspartate and L-Proline showed concentration-dependent fungal growth-promoting effects specifically under nitrogen limitation. For both metabolites, we predicted a net consumption by *C. albicans* growth inhibiting bacteria. It is noteworthy that, while we did not test for morphological changes, proline and putrescine are known inducers of hyphae formation in *C. albicans*, which may therefore be influenced by bacterial production and consumption of these metabolites [46–48]. Interestingly, desoxy-adenosine showed a growth-promoting effect at low concentrations, most pronounced under nitrogen limitation, whereas higher metabolite concentrations had negative effects on *C. albicans* growth. In our in silico model we predicted its production by *C. albicans* growth inhibting bacteria. A bacterial production of desoxy-adenosine may therefore lead to sufficiently high concentration levels of this metabolite that might restrict growth of the fungus. In summary, our in silico analysis of metabolite exchanges between fungus and bacteria and the shown experimental data provide a concept for predicting and testing potentially fungal growth modulating metabolites.

Next, we investigated whether metabolic interactions are the main driver of the observed abundance-based associations of gut bacteria and *C. albicans* by analyzing stool samples from a cohort of 24 cancer patients (Supplementary Table S8). We assessed the structure of the gut microbiome in samples via shotgun metagenomic sequencing, generating 118.1 Gbp of sequencing data with an average of 2 × 26.2 million paired-end reads per sample. Taxonomic profiling revealed that Bacteroidetes (54.3%) and Firmicutes (36.3%) were the most abundant phyla, followed by Proteobacteria (5.83%), Verrucomicrobia (1.46%) and Actinobacteria (1.34%). From the 400 bacterial species identified in our samples, we retrieved GSMMs for 247 (Fig. 3B, Supplementary Fig. S2). The mean relative abundance of *C. albicans* within the fungal community as revealed by ITS1 amplicon sequencing of all cancer patient samples was 39.7% over all patient samples, while individual samples covered a broad relative abundance range from 0.1% to 99.8% relative *C. albicans* abundance. Only five bacterial species had a relative abundance with a significant correlation by Spearman’s coefficient to *C. albicans* relative abundance across all patients (*Clostridium scindens* ρ = 0.45, *Hungatella hathewayi* ρ = 0.55, *Flavonifacfractor plautii* ρ = 0.46, *Barnesiella intestinihominis* ρ = −0.47 and *Alistipes putredinis* ρ = −0.42, Fig. 3B). Although the influence of external factors such as gender, age or therapy type cannot be completely ruled out, this suggests that, at least in terms of relative abundance compared to other fungal species, a limited set of gut microbes might influence *C. albicans* prevalence levels more than the remaining gut microbiome constituting bacteria. Of note, the directionality of the observed significant correlation was in accordance with the predicted paired-growth relationship by the GSMMs for all five bacteria (Supplementary Table S1). We also calculated Growth Rate InDex (GRiD) [49] to estimate in situ growth rates for bacteria species and analyze correlation to *C. albicans* relative abundance. We observed a finite GRiD value for 98 of the species in Fig. 3B (cf. Supplementary Table S9). GRiD values for two bacteria significantly correlated with *C. albicans* abundance (positive for *Roseburia inulinivorans* and negative for *Parabacteroides distasonis*, Fig. 3B) in the same direction as predicted by our GSMMs.

We next investigated to what extent fungal growth is influenced by spent media of selected bacterial strains for which we established cultivation protocols in our lab (Methods). Among the species selected for in vitro spent media experiments, *A. putredinis* had the strongest inhibitory effect in Western-diet conditions in in silico predictions (Supplementary Table S1). This species was also found significantly negatively correlated with *C. albicans* abundance in the human gut (Fig. 3B). Of note, adjusting for the external factors gender, age, ethnicity or immunotherapy application in our metagenomic dataset, affected this significant negative correlation only slightly (Supplementary Table S8). The addition of spent media from *A. putredinis* to *C. albicans* cultures (cf. Methods for details) resulted in up to 23% growth inhibition of the fungus (Supplementary Table S2). Interestingly, both butanoic and propanoic acid, two short chain fatty acids (SCFAs) with fungistatic properties produced by the gut microbiome [50, 51] were found with slight elevated concentrations in the spent media of *A. putredinis* (202 µM and 116 µM compared to 148 µM and 88 µM in modified GAM for propanoic and butanoic acid, respectively, Supplementary Table S2). *A. putredinis* is a reported producer of propionate presumably next to succinate [51]. Of note, for pairwise in silico simulations over all possible combinations of *A. putredinis* and any bacteria that are present in our human samples, we predicted that in 63% of the paired models *A. putredinis* can secrete propanoic acid. These data indicate that to a certain extend *A. putredinis* might contribute to global propanoic but also butanoic acid levels, two health promoting SCFAs [52]. In addition, *A. putredinis* is dominant in the fecal microbiota of healthy humans [53] and important for the maintenance of a healthy intestinal barrier [54]. Generally, the genus Alistipes shows disease protective effects against a number of diseases including fibrosis, colitis, cancer or cardiovascular disease [55]. Our results hint that *A. putredinis* is also potentially able to prevent elevated *C. albicans* levels, and we suggest more in-depth studies of *A. putredinis* in conjunction with *C. albicans*. To further examine whether the observed inhibition was a methodological artifact we performed additional experiments with species showing either positive or negative interaction with *C. albicans*. For example, our in silico predictions show that *Ruminococcus torques* contributes positively to *C. albicans* growth, while also a non-significant positive correlation between this species and *C. albicans* levels was observed from the metagenomics data (Fig. 3B, Supplementary Table S8). Indeed, in our spent media experiment we also observed a positive effect of *Ruminococcus torques* on *C. albicans* growth (15% for 100% spent media, 12% for 75% spent media, Supplementary Table S2). Other strains showed varied levels of *C. albicans* growth inhibition which is in agreement with our metagenomic and spent media data except for *Escherichia coli*. For *E. coli* we observe a disagreement between a predicted positive growth effect on *C. albicans*, a low positive correlation in the metagenomics data, and a negative effect in the spent media experiments. Interestingly, *E. coli* was found to produce a soluble fungicidal factor, which cannot be captured by our metabolic model and might explain the discordance to our spent media results [56].

To further support the idea that metabolic interactions with a few bacterial species might be sufficient to determine *C. albicans* colonization levels, we investigated, by ordinal regression analysis, if the ratio of abundance x growth coefficients for all bacterial inhibitors vs. promoters correlated with *C. albicans* levels (Methods, Ordinal regression model). No significant correlation was seen when considering all species (Fig. 3C, left), indicating that the use of all species as predictors does not allow for a good model to predict *C. albicans* levels. Of note, the ratio of inhibitors to promoters calculated using only species that were active in at least one patient sample according to GRiD was not significantly correlated to *C. albicans* abundance levels as well (Fig. 3C, right). The critical role of metabolic interactions between the limited set of 7 significantly correlating bacterial species identified above to *C. albicans* relative abundances was evident in the ordinal regression model we developed using the interaction growth coefficients and relative abundances of the bacteria. We investigated if GSMM computed growth coefficients, relative bacterial abundance, or both were good predictors for *C. albicans* levels (Method, Ordinal regression models). We obtained the highest performance of 0.78 Area Under the Receiver Operating Characteristics (AUROC) using growth prediction coefficients from our GSMM computation (Fig. 3D, left). Using only relative bacteria abundance resulted in 0.56 AUROC, whereas a model using both interaction coefficients and growth data resulted in 0.58 AUROC. In addition to using bacteria that show significant correlations to *C. albicans* relative abundances as model features, we filtered candidate bacteria by discarding first the bacteria prevalent in at least 10%, but not more than 90% of the samples. This reduces the number of feature candidates to 121 bacteria. We selected next 10 subgroups of our samples by ignoring ~10% samples in each subset, such that each sample was once not part of the subset. For each subset we started with one bacterium and assessed model performance consecutively by adding further bacteria until all bacteria were included as features. Bacteria that caused a drop in model accuracy or regression slope were discarded, followed by another iteration of the model performance evaluation. These steps were repeated until no performance-impairing bacteria were left in the feature list. The union of the gained feature candidates across all 10 subsets resulted in a feature list of 57 gut bacteria, which included, among others, *A. putridinis* (Supplementary Table S10). We evaluated the regression model with these bacteria and obtained 0.89 AUROC using only growth prediction coefficients from our GSMM computation (Fig. 3D, right). Using only relative bacteria abundance or both, interaction coefficients and growth data, resulted again in a performance drop down to 0.60 and 0.54 AUROC, respectively. These results demonstrate that GSMM analysis based interaction coefficients could be used as predictors of ordinally scaled *C. albicans* levels. Our results indicate that the intrinsic metabolic interactivity between a fungus and bacteria contains valuable information about the performance of classification models. This information should be more extensively applied in future classification studies.

In conclusion we expanded the concept of using in silico metabolic interaction calculations to accurately predict pairwise beneficial or detrimental effects on co-existing organisms [18, 25, 57] to bacterial-fungal interaction predictions based on studies suggesting that key gut species might determine beneficial outcomes in patients with a range of diseases [5, 6, 9]. Selected metabolite experiments and shotgun metagenomics sequencing back our in silico modeling concept based on pairwise metabolic interaction simulations. Further studies of our predictions for major metabolic pathways, for example for carbon compounds and amino acids, may elucidate the specific mechanisms of these influences. Selected metabolite measurements in defined media could be used further for accurate predictions of potential metabolite candidates that are preferentially used by e.g., gut microbes over *C. albicans* and can hint towards bacterial species that specifically secrete *C. albicans*-inhibiting metabolites. Taken together, our findings support that specific gut bacteria influence gut colonization by *C. albicans*. Moreover, our analysis indicates that it may be possible to design synthetic communities with only a few bacterial species that could then influence essential metabolic activities of *C. albicans* and prevent fungal overgrowth. Further refinements of our model including compartmentalization complemented by comprehensive single-knockout studies of bacteria or the fungus may further improve the predictive capacity. Also additional diets and growth media compositions beyond the three used in this study may be tested, since e.g., GAM compositions may vary, therefore influence in silico predictions and in general, might not reflect in vivo conditions as well as high fiber, western or other common human diets. Despite pairwise interactions were shown to be key drivers of the dynamics of microbial communities [58], additional in silico simulations of multiple interactions with e.g., the recently published MAMBO algorithm [59] need to be addressed, to potentially extend our understanding of the intricate relationship between *C. albicans* and the gut microbiota and its effect on *C. albicans* levels. Finally, tools that incorporate spatial information [60, 61] could determine the impact of niche colonization by gut fungal and bacterial species. In the present study we specifically focused on the intricate relationship between (gut) bacteria and *C. albicans* to elucidate their relationship independent of host factors in order to keep free parameters in a feasible range. Host factors are key modulators of fungal–bacterial or fungal–bacterial–host interactions as could be shown in other studies [3, 62–64]. Though adding considerable complexity to the setup, an extension of our conceptual approach to study specifically metabolic modes of fungal–bacterial interaction with host factors might play an important complementary role as long as the predictive capacity can be supported by sufficient data around the triangle host, bacteria and fungus. In summary, our strategy of studying fungal–bacterial relationships in the gut using an in silico, metabolism-driven approach already yielded promising results. Our approach adds another useful layer of in silico predictions that can contribute to stratify identification of potentially clinically relevant gut bacteria in face of the steadily growing amount of high throughput data. Ultimately, including metabolic in silico analysis could promote additional systems-biology and systems-medicine studies that focus on fungal infections and their often lethal implications to humans.

## Supplementary information

Supplementary Material

Supplementary Data S1

Supplementary Figure S1

Supplementary Figure S2

Supplementary Table S1

Supplementary Table S2

Supplementary Table S3

Supplementary Table S4

Supplementary Table S5

Supplementary Table S6

Supplementary Table S7

Supplementary Table S8

Supplementary Table S9

Supplementary Table S10
