# Supplementary material for: Metabolic modeling predicts specific gut bacteria as key determinants for Candida albicans colonization levels
Source: ISME J. 2020 Dec 15;15(5):1257–70. doi: 10.1038/s41396-020-00848-z (PMC8115155; doi:10.1038/s41396-020-00848-z)
Supplement: Supplementary file 1 — Supplementary Material [file 41396_2020_848_MOESM1_ESM.docx]

**Supplementary Material**

Legends for separate supplementary figures and tables; extended details for model reconstruction and simulation, mycobiome and metagenomics sequencing

Contents

[Separate supplementary figures and tables - Legends 2](#_Toc56248635)

[Model reconstruction 3](#_Toc56248636)

[Pairwise simulations 5](#_Toc56248637)

[Mycobiome sequencing 7](#_Toc56248638)

[Metagenomic sequencing 8](#_Toc56248639)

[References 9](#_Toc56248640)

## Separate supplementary figures and tables - Legends

Supplementary Data S1: *Candida albicans* genome-scale metabolic model

Supplementary Figure S1: Vector-based image version of Fig. 2C

Supplementary Figure S2: Vector-based image version of Fig. 3B

Supplementary Table S1: All *in silico* prediction results for individual and pairwise Flux Balance Analysis experiments

Supplementary Table S2: Results for spent media experiments, pH information and short chain fatty acid measurements

Supplementary Table S3: *Candida albicans* genome-scale metabolic model information - comprehensive list including all reactions and metabolites

Supplementary Table S4: All *Candida albicans* genome-scale metabolic model refinements

Supplementary Table S5: Comparison of model predictions and phenotypic microarray experiments on different substrates

Supplementary Table S6: GAM media composition and definition

Supplementary Table S7: *In silico* predictions for exchange reaction fluxes

Supplementary Table S8: Description of stool samples from human cohort and results for partial Spearman correlation of *A. putredinis* and *C. albicans*

Supplementary Table S9: Computed GRiD prediction values

Supplementary Table S10: Selected bacteria species as features for ordinal regression model

## Model reconstruction

To generate the *C. albicans* GSMM, we used the *C. albicans* metabolic model automatically reconstructed by the CoReCo pipeline as a template. In brief, CoReCo combines information from multiple data sources into a unified database and evaluates the probability of any reaction occurring in the target organism by computing a score for each enzyme based on sequence homology (1). We refined the model in four consecutive steps.

First, we identified duplicate metabolites. In this step, we identified compounds with of multiple synonyms, database IDs, or different naming conventions, e.g., generic and stereoisomeric names. In the latter case, the more specific isoform names were kept and generic names deleted. Affected reactions were refined accordingly (Table S3).

Second, we determined and resolved erroneous EGCs that created energy compounds such as ATP without the need of nutrient uptake using the Fritzemeier method (2). In brief, 13 dissipation reactions for energy-transmitting metabolites (e.g., ATP, NADPH) were defined and added to the model. Each dissipation reaction flux was maximized while prohibiting all model influxes. A positive optimal value indicated the minimum number of non-overlapping EGCs for each energy compound. The related flux distribution represented a set of active fluxes participating in EGCs. We applied pFBA (3) to pinpoint minimal sets of reactions causing EGCs. We interrogated the directionality of these active reactions based on BioCyc and corrected otherwise infeasible reactions (Table S3).

Third, we evaluated model phenotype accuracy using a set of experimental phenotypic microarray (Biolog) data for white and opaque *C. albicans* cells at 25°C and 37°C (4) and for data generated in our own lab for a prototrophic strain as well as mutant strains of *C. albicans* (cf. Phenotypic microarray experiments section). The model was examined for the ability to use 114 carbon, 74 nitrogen, 38 phosphate, and 16 sulfur sources. For the Ene et al. data *C. albicans* was considered to be able to grow on a source if it surpassed the growth signal of the control in at least one condition of four possibilities: white or opaque cells at 25°C or 37°C. Growth conditions were only considered if in agreement between the Ene et al. data and our results for the prototrophic strain.We resolved incompatibilities by manually curating network gaps based on BioCyc reaction information specific for *C. albicans* strain SC5314, or modified directionalities of existing model reactions to comply with the Biolog data (Table S3 and S4). For instance, according to the Biolog data, *C. albicans* can grow on d-amino-N-valeric acid as a nitrogen source (4), yet the model showed zero biomass formation for this condition. Also, D-proline can be produced through the reaction D-proline + NADH <=> NAD(+) + d-amino-N-valeric. However, no network component was present in the model that allowed reduction to the essential compound nitrogen via D-proline metabolism. To enable growth on d-amino-N-valeric acid we identified the proline racemase reaction as erroneously irreversibly converting L-proline to D-proline. Investigating this reaction in BRENDA and BioCyc showed that D- and L- forms of proline can reversibly interconvert. Altering the reaction directionality to be reversible state resolved this incompatibility.

Fourth, original exchange reactions were modified based on flux variability analysis (FVA) (5). Initially, we performed FVA for the original exchange reactions of the model. We removed exchange reactions that were prohibited to carry any flux and hence could not transport metabolites (exchange reactions with zero feasible flux). Finally, if not present, we added exchange reactions for metabolites (e.g., different carbon or nitrogen sources in the previous step) for which the Biolog data showed growth capability (Data S1, Table S2, S3).

## Pairwise simulations

Pairwise simulations adapted from Heinken and Thiele (6) were performed using 818 AGORA 1.03 GSMMs (7) downloaded from (<https://www.vmh.life>) and 92 CarveMe gut bacterial GSMMs (8). The *C. albicans* GSMM was paired to individual bacterial GSMMs by defining a common compartment for *C. albicans* and individual bacteria GSMMs. This compartment imitated a habitat such as the intestinal lumen or laboratory plates for both organisms, where metabolic interchange could occur (6). Coupling constraints were applied to associate the flux of all reactions for each model to its biomass formation, thereby avoiding zero flux through biomass formation when network fluxes were non-zero (9). The joined model was optimized by simultaneously maximizing *C. albicans* and bacteria biomass reactions. We ran pairwise simulations using pFBA simulating anaerobic conditions to resemble the gut environment. The interaction effect of each bacterial type on *C. albicans* and vice versa was measured using:

$$Y_{B\to CA}=log2\left( \frac{G_{CA, pair}}{G_{CA,indv}} \right) , Y_{CA\to B}=log2\left( \frac{G_{B,pair}}{G_{B,indv}} \right),$$

where Y_B→CA_ and Y_CA→B_ quantify the interaction effect of a bacterial type on *C. albicans* and *C. albicans* on the bacteria, respectively. G_CA,pair_ and G_CA,indv_ are optimized *C. albicans* growth rates reported in paired simulations with a given bacterial model and an individual *C. albicans* simulation, respectively. G_BS,pair_ and G_BS,indv_ are optimized bacterial growth rates in a paired simulation with *C. albicans* and as an individual model, respectively.

An organism was considered to be a promoter or inhibitor if the growth rate of the paired organism increased or decreased by at least 10% in the paired simulation compared to the individual growth rate. Six different ecological interaction types of mutualism, commensalism, amensalism, neutralism, parasitism, and competition were assigned based on whether bacteria promoted, inhibited or had neutral effects on *C. albicans* and vice versa.

To identify *C. albicans* reactions with pronounced flux differences in paired simulations with inhibiting and promoting bacteria, reactions were first filtered to show at least 60% consistent flux direction for each group separately. Reaction flux differences were then determined by calculating median flux values for each of the selected reactions across all *C. albicans* models for each group separately (*C. albicans* paired with inhibiting and *C. albicans* paired with promoting bacteria).

Model reconstruction and manipulations and linear programming tasks were in COBRApy (10) using Python 3.6.4 and the IBM ILOG CPLEX Optimizer (version 12.8). Addition of coupling constraints and performance of pairwise simulations was with CPLEX Python API. Statistical analyses used R, version 3.6.

## Mycobiome sequencing

Total DNA was extracted using the innuPREP Stool DNA Kit (Analytik Jena) following manufacturer´s instructions. After DNA concentration measurement on a NanoDrop (Thermo Fisher Scientific), 20ng of the DNA was used as input for PCR amplification of the fungal ITS1 region. The library construction was performed as described elsewhere (11), using the amplification primers ITS1f/ITS2, which were fused with golay indices and adapter sequences as shown in the following table:

| Forward construct | Construct | Sequence |
| --- | --- | --- |
|  | 5´Illumina adapter | AATGATACGGCGACCACCGAGATCTACAC |
|  | Forward linker | GG |
|  | Forward primer (ITS1f) | CTTGGTCATTTAGAGGAAGTAA |
| Reverse construct | Construct | Sequence |
|  | 3´Illumina adapter | CAAGCAGAAGACGGCATACGAGAT |
|  | Golay barcode | NNNNNNNNNNNN |
|  | Reverse linker | CG |
|  | Reverse primer (ITS2) | GCTGCGTTCTTCATCGATGC |

The PCR was performed on a S1000™ Thermal Cycler (BIORAD) in 50μl reactions using the Platinum™ PCR SuperMix (Thermo Fisher Scientific). Non-template controls were used to control potential contamination during the amplification process. Thermal conditions included an initial denaturation step (94°C for 3’), followed by 35 amplification cycles (94°C for 30’’; 52°C for 30’’;72°C for 60’’) and a final elongation step at 72°C for 10’. PCR product were purified using the NucleoMag NGS Clean-up kit (Macherey-Nagel) and quantified on D1000 Tapes using a TapeStation 2200 (Agilent Technologies). The libraries were equimolarly pooled and prepared for Illumina Sequencing using the MiSeq Reagent Kit v3 (Illumina) and following manufacturers’ instructions. Run plan and sequencing reagents and primers were adapted according to Caporaso et al. 2012 (12). Sequencing was performed on a MiSeq apparatus (Illumina) with 251 cycles.

## Metagenomic sequencing

Fecal samples were either frozen immediately at -80°C or briefly stored by participants in -20°C freezers before being transported to laboratory within 24 hours on ice. Fecal genomic DNA from human subjects was extracted as described in (13). All samples were sequenced on the Illumina HiSeq 4000 platform (Illumina San Diego, California, USA; Paired-end; insert size, 350 bp; read length, 150 bp) by BGI (Hong Kong S.A.R., China).

## References

1. Castillo S, Barth D, Arvas M, Pakula TM, Pitkänen E, Blomberg P, et al. Whole-genome metabolic model of Trichoderma reesei built by comparative reconstruction. Biotechnology for Biofuels. 2016;9:252.

2. Fritzemeier CJ, Hartleb D, Szappanos B, Papp B, Lercher MJ. Erroneous energy-generating cycles in published genome scale metabolic networks: Identification and removal. PLOS Computational Biology. 2017;13:e1005494.

3. Lewis NE, Hixson KK, Conrad TM, Lerman JA, Charusanti P, Polpitiya AD, et al. Omic data from evolved E. coli are consistent with computed optimal growth from genome-scale models. Molecular Systems Biology. 2010;6.

4. Ene IV, Lohse MB, Vladu AV, Morschhäuser J, Johnson AD, Bennett RJ. Phenotypic Profiling Reveals that *Candida albicans* Opaque Cells Represent a Metabolically Specialized Cell State Compared to Default White Cells. mBio. 2016;7.

5. Mahadevan R, Schilling CH. The effects of alternate optimal solutions in constraint-based genome-scale metabolic models. Metabolic engineering. 2003;5:264-76.

6. Heinken A, Thiele I. Systems biology of host-microbe metabolomics. Wiley Interdisciplinary Reviews: Systems Biology and Medicine. 2015;7:195-219.

7. Magnúsdóttir S, Heinken A, Kutt L, Ravcheev DA, Bauer E, Noronha A, et al. Generation of genome-scale metabolic reconstructions for 773 members of the human gut microbiota. Nature Biotechnology. 2016;35:81-9.

8. Machado D, Andrejev S, Tramontano M, Patil KR. Fast automated reconstruction of genome-scale metabolic models for microbial species and communities. Nucleic Acids Research. 2018;46:7542-53.

9. Heinken A, Sahoo S, Fleming RMT, Thiele I. Systems-level characterization of a host-microbe metabolic symbiosis in the mammalian gut. Gut Microbes. 2013;4:28-40.

10. Ebrahim A, Lerman JA, Palsson BO, Hyduke DR. COBRApy: COnstraints-Based Reconstruction and Analysis for Python. BMC Systems Biology. 2013;7:74.

11. Walters W, Hyde ER, Berg-Lyons D, Ackermann G, Humphrey G, Parada A, et al. Improved Bacterial 16S rRNA Gene (V4 and V4-5) and Fungal Internal Transcribed Spacer Marker Gene Primers for Microbial Community Surveys. mSystems. 2016;1:9-15.

12. Caporaso JG, Lauber CL, Walters WA, Berg-Lyons D, Huntley J, Fierer N, et al. Ultra-high-throughput microbial community analysis on the Illumina HiSeq and MiSeq platforms. ISME Journal. 2012;6:1621-4.

13. Qin J, Li Y, Cai Z, Li S, Zhu J, Zhang F, et al. A metagenome-wide association study of gut microbiota in type 2 diabetes. Nature. 2012;490:55-60.
