# Supplementary figures and images for: Metabolic modeling predicts specific gut bacteria as key determinants for Candida albicans colonization levels
Source: ISME J. 2020 Dec 15;15(5):1257–70. doi: 10.1038/s41396-020-00848-z (PMC8115155; doi:10.1038/s41396-020-00848-z)

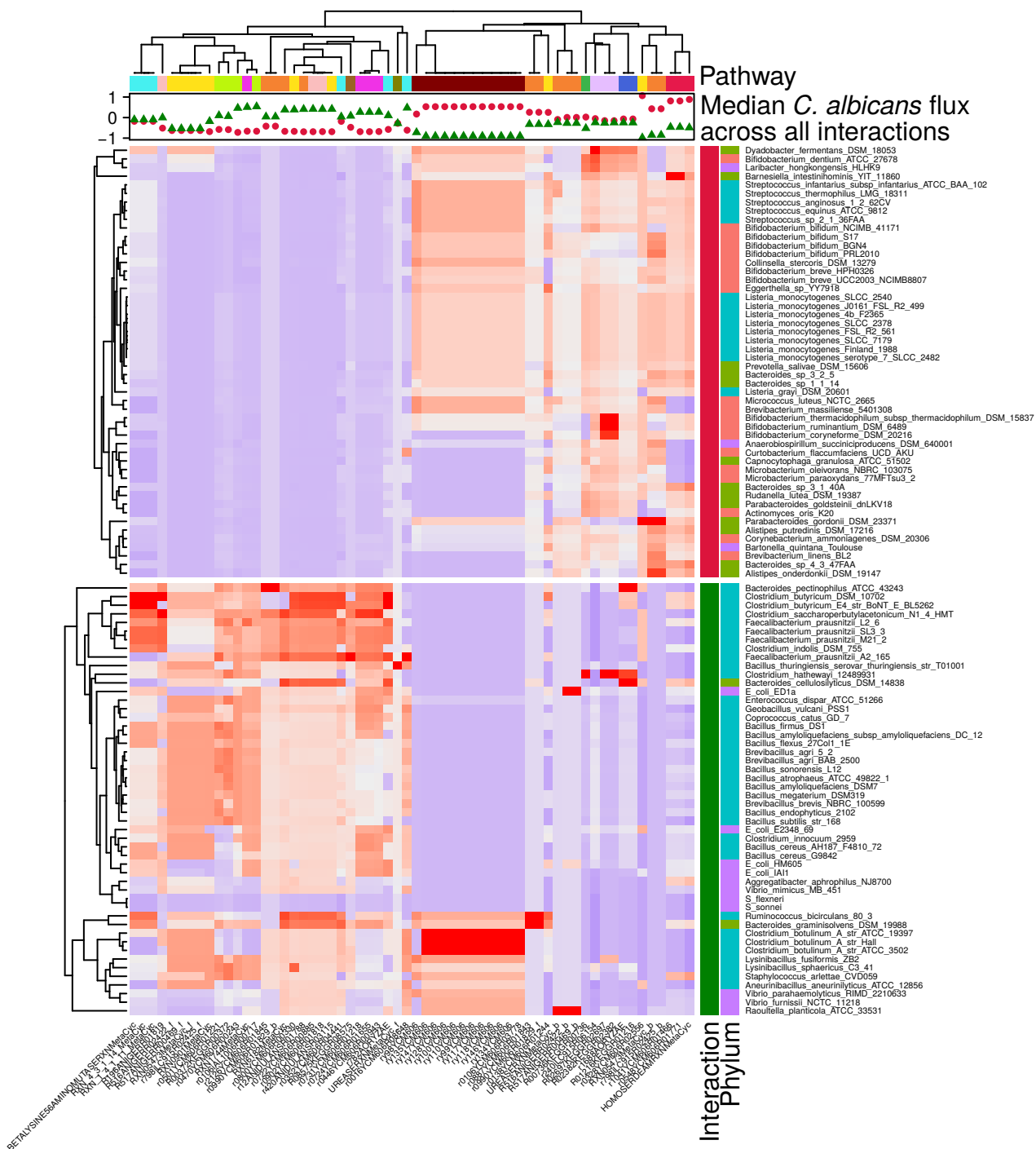

Flux value (z-score)

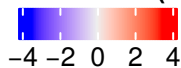

Interaction

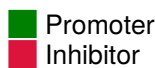

Phylum

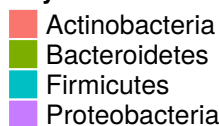

Pathway

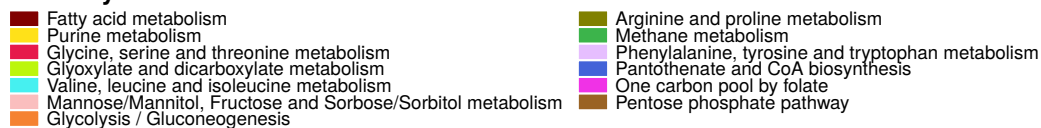

Supplement: Supplementary file 3 — Supplementary Figure S1 [file 41396_2020_848_MOESM3_ESM.pdf]
