# Supplementary material for: Metabolic modeling predicts specific gut bacteria as key determinants for Candida albicans colonization levels
Source: ISME J. 2020 Dec 15;15(5):1257–70. doi: 10.1038/s41396-020-00848-z (PMC8115155; doi:10.1038/s41396-020-00848-z)
Supplement: Supplementary file 4 — Supplementary Figure S2 [file 41396_2020_848_MOESM4_ESM.pdf]

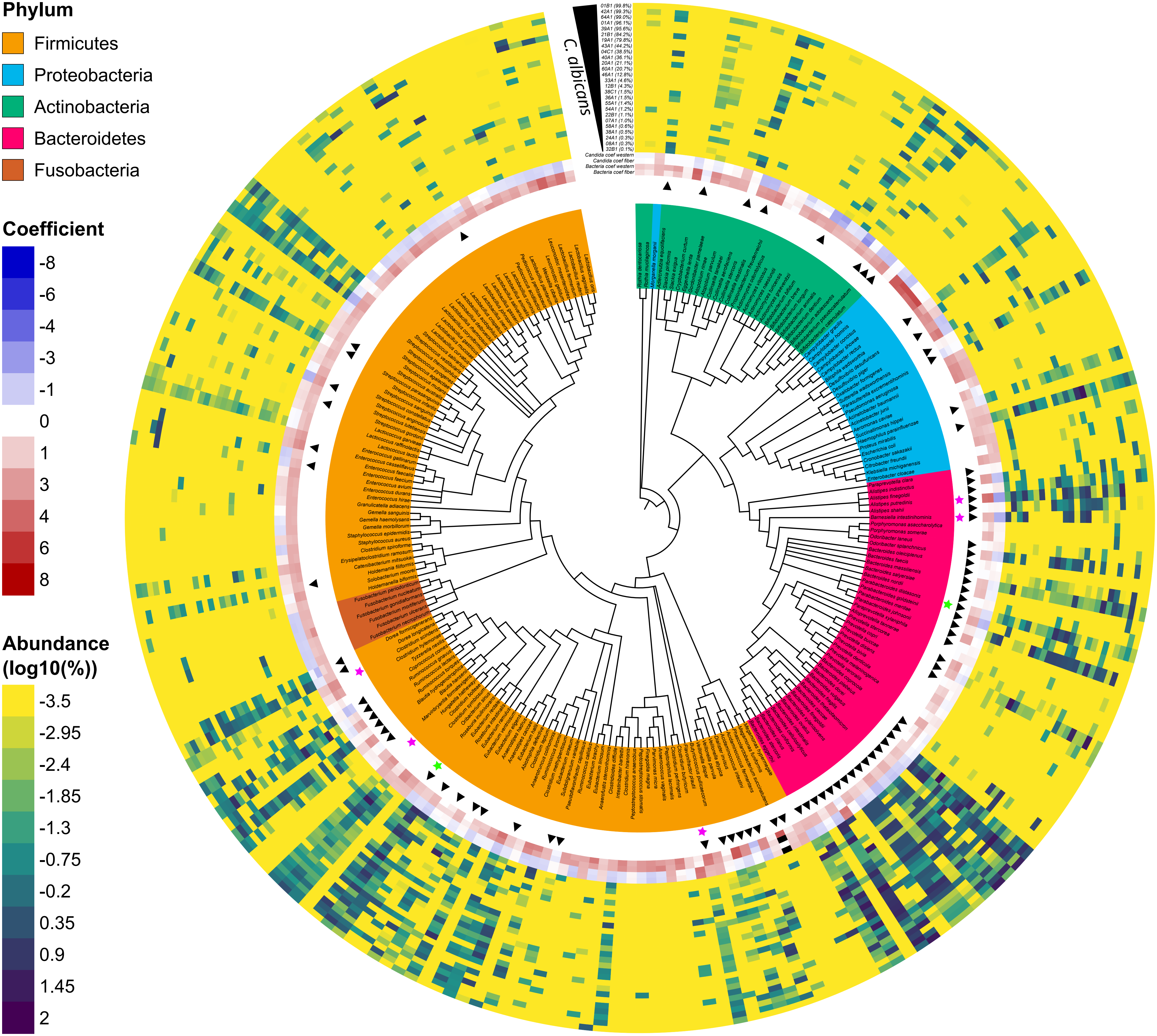

Phylum
Firmicutes
Proteobacteria
Actinobacteria
Bacteroidetes
Fusobacteria
Coefficient
-8
-6
-4
-3
-1
0
1
3
4
6
8
Abundance (log10(%))
-3.5
-2.95
-2.4
-1.85
-1.3
-0.75
-0.2
0.35
0.9
1.45
2
C. albicans
Candida coef western
Candida coef fiber
Bacteria coef western
Bacteria coef fiber
